# Implementation of Behavior Change Theories and Techniques for Physical Activity Just-in-Time Adaptive Interventions: A Scoping Review

**DOI:** 10.3390/ijerph22071133

**Published:** 2025-07-17

**Authors:** Parker Cotie, Amanda Willms, Sam Liu

**Affiliations:** School of Exercise Science, Physical and Health Education, University of Victoria, Victoria, BC V8W 2Y2, Canada

**Keywords:** physical activity, digital health, mobile health, just-in-time adaptative interventions

## Abstract

(1) Background: Physical activity (PA) is a key modifiable risk factor for chronic diseases, yet many adults do not meet PA guidelines. Just-in-time adaptive interventions (JITAIs), a type of mobile health (mHealth) intervention, offer tailored support based on an individual’s context to promote PA. Integrating behavior change techniques (BCTs) and theories is critical to the design of effective mHealth interventions. Understanding which BCTs and theories work best can inform future JITAI development. (2) Objective: The objective of this study is to examine how behavior change theories and BCTs are implemented in mHealth PA JITAIs and assess their relationship with PA-related outcomes. (3) Methods: This scoping review followed the PRISMA-ScR guidelines. A total of 29 studies were included. (4) Results: The most commonly used BCTs include prompts/cues (*n* = 29), goal-setting (behavior) (*n* = 15), and feedback on behavior (*n* = 14), while self-determination theory (*n* = 4) and social cognitive theory (*n* = 4) are the most commonly used theories. However, there is insufficient evidence as to which theories and BCTs are most effective in eliciting effective PA behavior change. (5) Conclusions: Clearer reporting and integration of BCTs and behavior change theories, along with optimized user interfaces, are needed to improve the intervention quality, replicability, and long-term effectiveness of PA JITAIs.

## 1. Introduction

Chronic diseases, such as cardiovascular disease, chronic respiratory diseases, and diabetes, are the leading causes of death worldwide, responsible for 39 million (72%) of the total global deaths in 2021 [1]. Physical activity (PA) has been shown to be a key modifiable risk factor for many chronic conditions and can serve as a powerful lifestyle strategy for both management and prevention of chronic disease [2]. Despite these well-known benefits, almost one-third of adults worldwide do not meet the recommendations of at least 150 min of moderate-intensity PA or 75 min of vigorous-intensity PA per week [3]. While in-person PA interventions can be an effective option for combating inactivity, they often face challenges related to accessibility, cost, and scalability, particularly when targeting general populations [4,5]. In response, there is a growing need for innovative, scalable, and personalized approaches to support PA among adults not currently meeting PA guidelines.

The widespread adoption of smartphones has created new opportunities to deliver PA promotion interventions. In Canada and the United States, over 84% of the population now owns a smartphone, and an estimated 60% of the world owns a smartphone [6]. This increase in digital connectivity has supported the growth of mobile health (mHealth) interventions, which offer scalable and personalized solutions for promoting PA. Recent technological advancements of smartphones combined with wearable technology have also enabled a new type of mHealth intervention known as a just-in-time adaptive intervention, or a JITAI. These interventions use real-time data from wearable sensors to adapt the timing and content of support based on an individual’s current context and state. This allows the intervention to be delivered at moments when the individual is both in need of support and most likely to respond positively [7]. JITAIs are a novel method of delivering mHealth interventions, which adapt the support provided based on the individual’s status and context to deliver the intervention not only when the individual needs it but when they will be most receptive to the intervention [8]. Previous systematic reviews have already shown that JITAIs are more effective than non-JITAIs (g = 0.868, 95% CI = 0.41, 1.32) [9]. Although JITAIs show considerable promise, there is limited evidence on how behavior change theories and techniques have been applied in PA interventions.

Behavior change theories provide a conceptual framework for understanding behavior change. They help identify the mechanisms of action (e.g., motivation, self-efficacy, habit) that underpin behavior change [10]. JITAIs offer dynamic support by leveraging insights from traditional theoretical frameworks. For instance, JITAIs can foster autonomous motivation by delivering timely prompts that align with an individual’s goals, values, and sense of volition, which align with Ryan and Deci’s self-determination theory [11,12]. From a social cognitive perspective, JITAIs can enhance self-regulation by offering real-time feedback and support when individuals are most in need of reinforcement [13]. In turn, behavior change techniques (BCTs) are the observable components of interventions that translate theoretical constructs into practical strategies [14]. Examples of these practical strategies that can be implemented to change behavior include goal-setting, self-monitoring, or providing feedback. Overall, behavior theories guide the selection of appropriate BCTs, which in turn operationalize those theories within behavior change interventions. Thus, understanding how behavior change theories and techniques have been used in PA JITAIs, as well as which theories and techniques are effective, can provide important information for the design of future PA JITAIs.

In addition, there is a lack of reviews on how adaptive algorithms are designed and implemented in JITAIs. These algorithms determine how interventions respond dynamically to user data by adjusting the timing, type, and content of support based on behavior theories and techniques. For example, some JITAIs tailor prompts based on real-time PA levels, user goals, time of day, or contextual features, such as weather [15]. Others incorporate features such as recommender systems based on user preferences [16] or artificial intelligence (AI)-driven chatbots that allow for conversational support [17]. While these approaches reflect increasing sophistication, the underlying logic, decision rules, and data inputs used to guide personalization are often poorly described. This lack of transparency hinders efforts to evaluate, replicate, and optimize JITAI design.

Therefore, the objectives of this review are (1) to describe the implementation of behavior change theories, techniques, and the JITAI personalization algorithms in JITAIs designed to promote PA, and (2) to explore whether the use of certain theories and techniques in JITAIs led to greater improvements in PA outcomes.

## 2. Materials and Methods

This scoping review was conducted following the Preferred Reporting Items for Systematic Reviews and Meta-Analyses guidelines extension for scoping reviews (PRISMA-ScR) Checklist [18] and completed using Covidence software [19]. The completed PRISMA-ScR checklist is available in Appendix A (Table A1). The review protocol was pre-registered on the Open Science Framework prior to publication. Due to the nature of conducting a scoping review, ethics approval was not required for this work. We conducted an electronic literature search of Web of Science (EBSCO), MEDLINE (Ovid), PsycINFO (EBSCO), and SPORTDiscus (EBSCO). The search strategy consisted of three searches focusing on mHealth, JITAIs, and PA, respectively, which were combined to provide our results. The mHealth search string consisted of terms such as “mobile health”, “smartphones”, and “Apple Watch”. The JITAI search string consisted of terms such as “just-in-time”, “dynamic tailor”, and “real-time intervention”. An example search strategy and its results for PsycINFO (EBSCO) are available in Appendix B (Table A2). Additionally, reference lists were searched from relevant identified articles to detect any articles that were missed by our search strategy.

### 2.1. Eligibility Criteria

Studies deemed sufficient for further review from the search results had their titles and abstracts screened independently by the two authors (P.C., A.W.) to make exclusions. Our search strategy was executed in November 2024. Studies were selected for full-text review if they met all the following criteria: published in a peer-reviewed journal, described or used a mobile-based PA JITAI, and published in English. There was no restraint in terms of the date range for studies included in our literature search. Types of studies that met the criteria include protocol studies, intervention studies, randomized controlled trials, rationale and design studies, micro-randomized trial studies, participatory development studies, pilot studies, experimental studies, and feasibility studies. Studies that met these criteria were then entered into the full-text review, where they were thoroughly analyzed to determine whether they would be included in the review. Discrepancies in included texts by each author were settled through discussion between P.C. and A.W. at both the title and abstract screening stage, and the full-text review stage. In all instances, consensus was reached through discussion, and there were no unresolved disagreements that required arbitration. As a result, a third reviewer was not needed.

### 2.2. Data Extraction

We extracted the following characteristics from the selected studies into a spreadsheet for further analysis: (1) BCTs stated (BCTs explicitly stated within a study by the author), (2) BCTs implied (two authors (P.C., A.W.) independently identified BCTs implied in studies by aligning if the author descriptions of intervention descriptors aligned with the BCT taxonomy [20]; both reviewers compared coding results and resolved discrepancies through discussion until consensus was reached), (3) behavior change theory, (4) JITAI decision tree or algorithm, (5) description of JITAI, (6) intervention length, (7) desired outcome, (8) findings, (9) PA description, and (10) comparator. All data extracted from the review are available in Appendix C (Table A3).

### 2.3. Data Synthesis

We chose to conduct a scoping review rather than a systematic review or meta-analysis due to the variation in PA measurements and outcomes used by researchers. For our analysis, we initially identified and compiled a list of behavior change theories and techniques that had been implemented for JITAI PA interventions, their prevalence amongst the reviewed literature, and how these features had been delivered using mHealth tools. The behavior change technique taxonomy v1 from Michie and colleagues was used as our guide for BCTs [20].

To further explore the implementation of BCTs, we explored BCT combinations, analyzing how specific combinations of BCTs co-occur within interventions [21]. This approach recognizes that BCTs are often not applied in isolation but in conjunction with others, potentially enhancing intervention effectiveness. Upon extracting individual BCTs from each study, we identified pairings through frequency counts where two BCTs were deliberately integrated to influence PA behavior. To assess the effectiveness of individual BCTs and their pairings, we examined their association with significant improvements in PA outcomes, such as increased PA time or step counts. This analysis aimed to identify which BCTs and combinations thereof were linked to positive behavioral changes.

Furthermore, we conducted a descriptive analysis of the design and implementation of adaptive algorithms within JITAIs, which sought to determine how interventions respond dynamically to user data by adjusting the timing, type, and content of support based on behavior theories and BCTs. We extracted information on decision points, tailoring variables, intervention options, and decision rules from each study, where available.

## 3. Results

### 3.1. Study Selection

**Figure 1:**
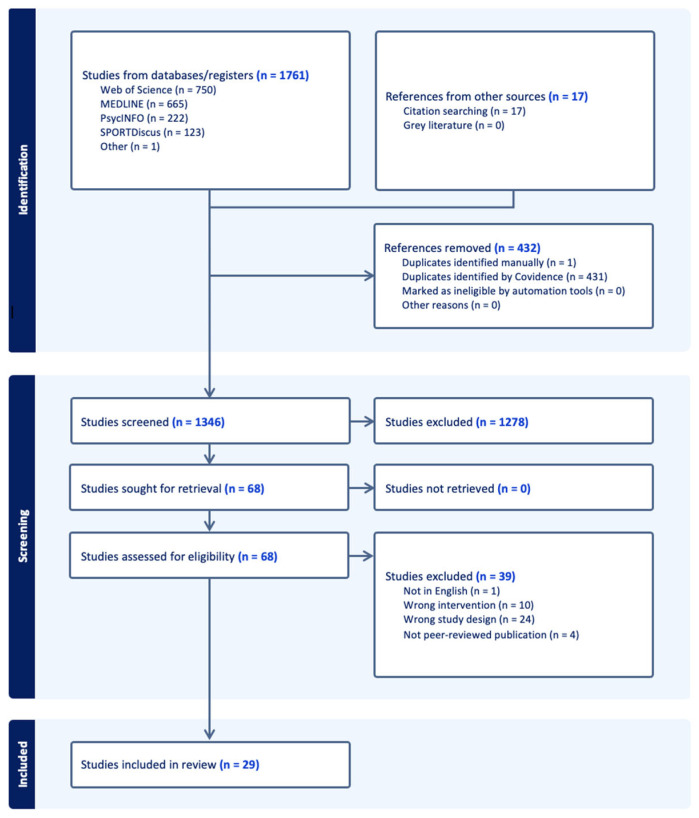
Preferred Reporting Items for Systematic Reviews and Meta-Analyses (scoping review) flowchart.

As shown in Figure 1, 1778 studies were imported for screening, 1761 from databases/registers and 17 from other sources. Of those 1778 studies, 29 met all inclusion criteria and were included in the final review.

### 3.2. Study and Participant Characteristics

Of the 29 studies included, 12 were from the United States, 6 were from the Netherlands, 2 were from Australia, 2 were from Germany, 2 were from the Czech Republic, and 1 each was from Belgium, Lebanon, Singapore, Switzerland, and Qatar. In terms of study design, 12 studies were feasibility or pilot studies, 6 were study protocols, 4 were randomized controlled trials, 4 were development studies, 3 were quasi-experimental studies, and 2 were micro-randomized trials. Of the 29 studies, 14 reported PA outcomes, with 8 employing within-person designs and 6 between-person designs. Studies that did not report PA outcome included study protocols, development studies, and some feasibility studies that did not report preliminary efficacy on behavioral outcomes. The average intervention length was 12.98 weeks (±14.7 weeks), with intervention lengths ranging from 1 week to 12 months. Further study characteristics are available within Appendix C.

Of the studies that recruited participants (*n* = 24/29, 82%), 13 recruited healthy but inactive adult participants, 4 studies recruited participants with diabetes, 2 studies recruited families, 2 studies recruited participants recovering from spinal cord injuries, 2 studies recruited older adults, 1 study recruited participants with hypertension, 1 study recruited participants with cardiovascular disease, and 1 study recruited participants recovering from metastatic gastrointestinal cancer surgery. The average sample size was 55.83 participants (±76.37 participants), with sample sizes ranging from 7 to 274 participants.

### 3.3. The Implementation of Behavior Change Theories and Techniques in PA JITAIs

Of the 29 papers included in the review, 16 explicitly stated a behavior change theory in which their JITAI was grounded. The most commonly stated behavior change theories were self-determination theory [11,12] (*n* = 4, 14%) and social cognitive theory [22] (*n* = 4, 14%). The next most common were Fogg’s behavior model [23] (*n* = 3, 10%) and the capability, opportunity, motivation–behavior (COM-B) model [24] (*n* = 3). Cialdini’s 7 Principles of Influence [25] (*n* = 1, 3%), the health action process approach [26] (*n* = 1, 3%), learning theory [27] (*n* = 1, 3%), Locke and Latham’s goal-setting theory (*n* = 1, 3%), Marlatt’s relapse-prevention model [28] (*n* = 1, 3%), the transtheoretical model [29] (*n* = 1, 3%), Rothman’s theory [13,30] (*n* = 1, 3%), and self-regulation theory [13] (*n* = 1, 3%) were each stated once.

**Figure 2:**
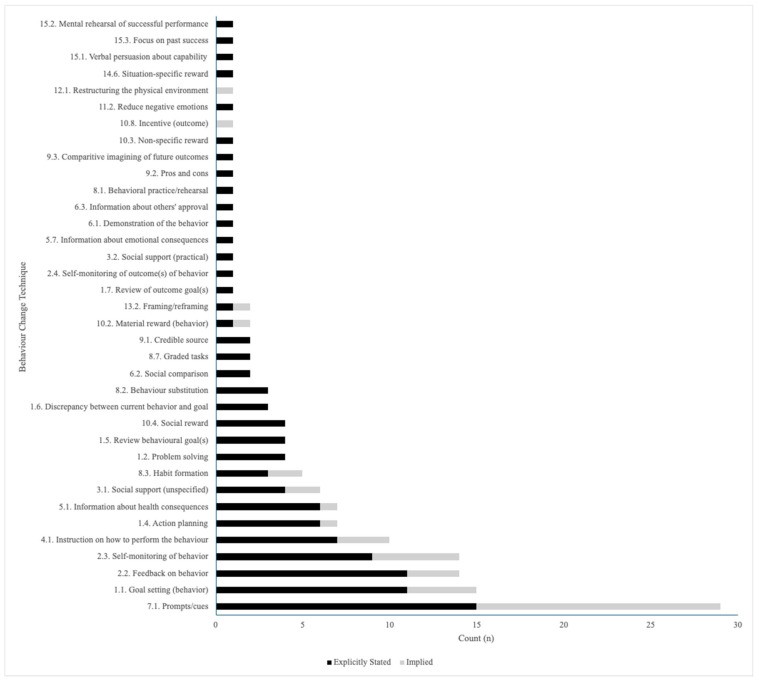
Behavior change techniques (BCTs) and their inclusion amongst studies.

A total of 36 unique BCTs were used across the reviewed literature. Of the 36 total BCTs used, most (34/36, 94%) of the BCTs were stated by the authors of the papers. Of the 36, 13 (36%) BCTs were implied, and were mapped onto the BCT taxonomy by the authors of this review (P.C., A.W.). As shown in Figure 2, the most commonly used BCT that was used in all studies (*n* = 29, 100%) was prompts/cues, due to the nature of PA JITAIs requiring prompts to perform activity. Other prominent BCTs included goal-setting (behavior) (15/29, 52%), feedback on behavior (14/29, 48%), self-monitoring of behavior (14/29, 48%), and instruction on how to perform the behavior (10/29, 34%).

To gain a deeper understanding of how BCTs are implemented in PA JITAIs, we analyzed the various ways BCTs are combined within these interventions. The BCT prompts/cues were not included in the pair frequency counts as prompts serve as the trigger for delivering a JITAI, rather than a component paired with other techniques. The most paired BCTs were goal-setting (behavior) and feedback on behavior (13 instances). For example, Carey and colleagues’ protocol for a JITAI among patients with spinal cord injury combined the use of behavioral goal-setting and feedback on behavior through daily goal-setting using a mobile app, combined with just-in-time tailored PA feedback messages framed within the COM-B model [31]. Another instance of this pairing was in a PA and diet JITAI for blood pressure reduction [32]. A total of 486 participants who self-reported having hypertension had the opportunity to set behavioral goals relating to choosing low-salt foods and step goals. In response to these goals, participants received personalized, adaptive behavioral feedback relating to these goals [32], for example, “The snow is not going to shovel itself! That is a lot of exercise right there, [Name]!” [32]. The second most prevalent BCT pairing was goal-setting (behavior) and self-monitoring of behavior (11 instances). An example of this pairing was shown through the use of an app which included an artificial intelligence health coach that interacted with the user through a chat-like feature [33]. Participants were encouraged to set behavioral goals, such as exercise minutes, and through the chat feature, the app would share figures of daily exercise to promote self-monitoring. Another example of this pairing was in the VALENTINE study where participants used an app that allowed for goal-setting and activity-tracking [34]. Additionally, participants were encouraged to wear an Apple Watch or Fitbit, which enabled self-monitoring of PA [34].

Another BCT combination prevalent in the literature was feedback on behavior and self-monitoring of behavior (nine instances). Daryabeygi-Khotbehsara and colleagues reported delivering these BCTs through in-app notifications from their iMove app designed for adults with type 2 diabetes [35]. Examples of notifications relating to these BCTs include “Congratulations! You achieved your daily physical activity goal [≥VALUE minutes] today. Keep up the good work!” to promote feedback on behavior [35]. An example of a self-monitoring of behavior notification developed for this app was “Check how much time you spent in physical activities yesterday.” [35]. Another example of the use of this BCT pairing was demonstrated by Hietbrink and colleagues who incorporated these BCTs to promote self-regulatory processes from the concept of negative feedback control [36]. In their E-supporter 1.0 app, feedback on behavior notifications was tailored based on meeting or failing to meet a weekly step goal [36]. Self-monitoring was included in the app through self-reported activities and a digital food diary, along with a Fitbit [36].

Goal-setting (behavior) with instruction to perform the behavior and self-monitoring of behavior with instruction to perform the behavior were each reported in combination in eight studies. In the ENERGISED app, participants were encouraged to set behavioral goals through phone counseling and received instructions on how to perform the behavior through educational leaflets [37]. The SNapp app is another example of this pairing where participant received messages encouraging users to set a daily step count goal for the upcoming week (i.e., “What’s your new goal, [NAME]? Set yourself an achievable walking goal for this week.”) to promote behavioral goal-setting [38]. Instruction to perform the behavior was encouraged through messages informing participants when they were near a green space to participate in PA (i.e., “Do you want to get some extra steps in today? You are close to a [green space type] where you can enjoy a nice walk.”) [38]. Examples of the self-monitoring of behavior and instruction to perform the behavior pairing were displayed in the Ally app [39] and the PAUL app [40]. Kramer and colleagues designed self-monitoring prompts to remind participants of their daily step goal, compare the participants’ current step count to their daily goal, and provide an estimate of walking minutes necessary to reach the goal [39]. In addition to these self-monitoring prompts, participants would receive instruction on how to perform a walking behavior randomly between 10 a.m. and 6 p.m. [39]. In the PAUL app, Sporrel and colleagues (2022) visually displayed the percentage of the PA goal that was met in the app to promote self-monitoring [40]. Additionally, in the app, participants received location-based exercise prompts with instructional videos in the direct environment of the user (i.e., if the participant was at the park, they would receive a video of how to do a push up in a park and use their surroundings to modify the exercise) [40].

### 3.4. JITAI Adaptive Algorithms

While each intervention had its own unique JITAI personalization algorithms that were used, there were some similarities seen across the reviewed literature. Personalization was also seen within the content of notifications, with one paper tailoring notifications based on PA level, goal, time of day, and weather [41]. Another prevalent feature was sedentary behavior interruption, with prompts encouraging movement to break up device-measured sedentary bouts [42,43]. Personalized coaching and content were amongst the most prevalent components seen across JITAIs. Coppens and colleagues (2024) implemented this by using a recommender system algorithm, which automatically generated personalized suggestions based on users’ preferences [16]. Activity-tracking and self-monitoring were other commonly seen features. Many JITAIs tracked measures such as steps, activity time, or metabolic equivalents (METs), a quantification of metabolic activity to measure PA, and presented users with graphs and data to allow for self-monitoring of their behavior and goal-tracking. Finally, while less prevalent than the other features, a feature worth noting is the integration of generative AI chatbots by both Kramer and colleagues and Vandelanotte and colleagues, which allowed participants to ask PA-related questions [17,39].

Despite these advancements, a significant concern is the insufficient reporting of the underlying algorithms that drive these personalization strategies. This lack of transparency limits the ability to assess the effectiveness of different algorithmic approaches and to guide the design of future interventions. Furthermore, many studies rely on static decision rules, such as if-then statements, without incorporating adaptive mechanisms that respond to changes in user behavior or context [44]. The absence of detailed reporting and adaptive algorithms reinforces the need for standardized guidelines to enhance the transparency and adaptability of JITAI designs.

### 3.5. Changes in PA-Related Outcomes for JITAIs

Only 14 of the 29 reviewed studies reported PA outcomes, due to many studies being protocol or feasibility studies, mainly reporting on outcomes other than behavior (i.e., recruitment, engagement, acceptability). Regarding specific PA outcomes, of six studies measuring steps, two demonstrated significant within-group improvements, but none showed between-group differences, relative to control. For light PA or MVPA time (*n* = 4/14, 29%), only one study reported significant within-group increases, with no between-group effects. Among two studies measuring METs, one showed significant within-group improvement; none reported between-group differences. Two studies assessing minutes spent walking showed no significant changes within or between groups. Most studies (13/14, 93%) utilized device-based PA measurement, with only one relying on self-reporting. Table 1 displays the PA outcome data.

Significant improvements in PA outcomes were observed in 5 of these 14 studies. We did not observe a clear pattern linking specific theories to improved PA outcomes; however, certain BCTs, particularly prompts/cues, self-monitoring, and goal-setting, were common in interventions that elicited significant PA results. Of these five studies, only two studies reported the behavior change theory used. These theories included learning theory, social cognitive theory, Fogg’s behavioral model [52], and self-determination theory [47]. In contrast, greater consistency was noted in the BCTs employed: prompts/cues [34,42,47,52,53], self-monitoring of behavior [34,52], behavioral goal-setting [34,47], instruction on how to perform the behavior [47], and providing information about health consequences [47] were prevalent BCTs among effective studies.

Among the studies that did not report statistically significant improvements in PA outcomes (*n* = 9, 64%), there was considerable variability in the use of theoretical frameworks, with no single theory used consistently across studies. Reported theories included Fogg’s behavioral model [40,46], goal-setting theory [46], a transtheoretical model [46], social cognitive theory [48], self-determination theory [54], and social influence strategies based on Cialdini’s principles [43], while several studies did not report any guiding theory [46,50,51,52]. Despite the lack of common theoretical grounding, a range of BCTs were applied. Prompts/cues (9/9, 100%) [40,43,45,46,48,49,50,51,54], goal-setting (behavior) (6/9, 67%) [40,43,45,46,48,54], and self-monitoring of behavior (5/9, 56%) [40,48,49,50,54] were the most frequently used BCTs, followed by feedback on behavior (4/9, 44%) [40,45,48,54] and instruction on how to perform the behavior (3/9, 33%) [40,48,54]. Examples of additional techniques included material rewards [40,49], social support [43], and habit formation [43].

## 4. Discussion

This scoping review aimed to describe the implementation of behavior change theories, techniques, and personalization algorithms in JITAIs targeting PA and to evaluate the relationship between the use of theories and BCTs in improving PA outcomes. Our findings reveal considerable variability in how theories and techniques are reported and operationalized in mHealth-based JITAIs, and limited evidence to support the association of behavior change theories and techniques with significant PA improvements.

In addressing the first objective, our results suggested inconsistencies in the implementation and reporting of behavior change theories and techniques. Only 55% of studies referenced a theoretical framework, most commonly self-determination theory and social cognitive theory. However, these theories were often only mentioned without clearly informing the intervention design, tailoring strategies, or logic models. Similarly, only two-thirds of studies reported using BCTs, with prompts/cues, goal-setting (behavior), and feedback on behavior being the most frequently applied. These BCTs are foundational in behavior change interventions but were seldom embedded in a broader, theory-informed strategy. Several well-established BCTs that support habit formation, motivation, and behavioral regulation, such as problem-solving or review of behavioral goals [55], were often not included.

The personalization algorithms central to JITAIs were also underreported. Only a subset of studies described their adaptation strategies in terms of tailoring variables and delivery conditions [16,17,32,34,35,38,41,45,47,50,51,52,56]. While some interventions detailed contextual inputs (e.g., location, time of day, or step count) or cited the use of decision rules ([32,34,36,38,41,46,47,50,51]), most did not comprehensively report the logic or algorithms guiding JITAI delivery. This gap limits the ability to replicate or evaluate the adaptiveness of interventions. The mHealth Evidence Reporting and Assessment (mERA) checklist developed by Agarwal et al. emphasizes the importance of transparent reporting of digital intervention components, including software platforms and adaptation protocols [57]. However, many studies in this review did not meet these standards.

The second objective of this review was to evaluate whether the presence of behavior change theories or techniques corresponded to improved PA outcomes. Of the 14 studies reporting PA outcomes, only 5 demonstrated statistically significant improvements. Among these, goal-setting (behavior) and self-monitoring were each used in two of the five studies, and instruction on how to perform the behavior and information about health consequences were each used in one study. However, three of the five studies successful in improving PA outcomes did not report using a behavior change theory, while the others referenced self-determination theory, learning theory, or Fogg’s behavioral model. This reporting is consistent with Hardeman and colleagues’ systematic review which also reflected on the underreporting of behavior change theories among PA JITAIs [15].

There are several reasons that may explain the limited number of statistically significant PA studies. Most interventions had short durations, with 12 of the 14 studies that reported PA outcomes lasting less than 10 weeks. This may be insufficient for behavior change to consolidate into a habit, especially given that 10 weeks is often cited as the minimum threshold for habit formation [58]. Additionally, 8 of the 14 studies had small sample sizes (n < 30), limiting statistical power and the generalizability of findings [59]. Many of the included studies were pilot or feasibility trials, which, while essential for early-stage testing, are not typically designed to assess intervention effectiveness [60].

Another concern was the underreporting of user interface design and user engagement strategies. While some studies such as Hietbrink et al. and Ismail & Al Thani described appealing and user-friendly apps, the majority did not provide visual documentation or discuss the usability of their platforms [36,49]. User interface quality is a critical determinant of digital intervention success, and optimized interfaces improve user engagement, a key mechanism through which JITAIs may influence behavior [61,62]. In our review, there was only a limited number of studies providing details on app design, and thus it remains unclear how interface design may have impacted study results.

Emerging work highlights the potential for AI to support more dynamic and scalable JITAIs. For example, Kramer et al. used the MobileCoach platform to develop a chatbot (Ally) that delivered prompts and incentives [39]. Vandelanotte et al. used natural language processing and Google’s Dialogue Flow to create an interactive chatbot for answering PA-related questions [17]. Willms & Liu explored using generative AI (ChatGPT) to rapidly generate PA JITAI content [63], while Haag et al. tested GPT-4 for adaptive decision-making within interventions [64]. These innovations represent promising directions for the field, although further research is needed to determine their long-term effectiveness, acceptability, and ethical implications.

Several implications for research and practice emerge from these findings. First, future JITAIs should be firmly grounded in behavior theory, with clear rationale for the selection and application of BCTs and BCT combinations. Reporting guidelines such as the BCT taxonomy [20] and the mERA checklist [57] should be routinely followed to enhance transparency and reproducibility. Second, adaptive mechanisms should be fully described, including the decision rules and contextual data that guide real-time content delivery. Third, app interface design deserves careful prioritization and optimization. Engaging, user-friendly, and intuitive platforms are critical for maintaining user retention and supporting intervention adherence over time. In this context, recent advances in “no-code” mHealth app development tools provide promising avenues to accelerate the design and testing of sophisticated JITAI. These tools allow researchers to build, evaluate, and optimize JITAI aimed to promote PA [65,66]. Finally, researchers should design studies with sufficient duration and sample size to detect meaningful changes in PA, while accounting for the diverse populations and behavioral targets involved.

This review has limitations. The included studies were highly heterogeneous in their designs, target populations, and outcome measures, which limited comparability. Reporting gaps made it difficult to definitively assess the influence of specific theories or techniques on outcomes. Moreover, a standardized measure of PA was lacking across studies, and some interventions measured only walking rather than total PA. The inclusion of many early-phase studies also constrained the generalizability of the findings. Additionally, we did not conduct a formal quality assessment, as this review included study protocols and exploratory research, which are not always suitable for traditional appraisal tools.

## 5. Conclusions

In conclusion, the use of behavior change theories and techniques in JITAIs for PA remains inconsistent and often underreported. While a small number of studies demonstrated significant improvements in PA, there was no clear pattern linking specific BCTs or theories to effectiveness. Transparent reporting of adaptive features, careful integration of theory, and attention to app usability will be critical for the continued development of effective and scalable JITAIs. Advancing these areas will help ensure that JITAIs fulfill their potential as innovative tools for promoting sustained PA in diverse populations.

## Figures and Tables

**Table 1:** Reported physical activity outcomes and intervention details.

Author, Year	PA Outcome	PA Measurement Tool	Length	Significant Results	Comparator
Boerema et al., 2019 [45]	PA Intensity	ProMove 3D Activity Sensor	1 week	No significant PA results	Baseline
Ding et al., 2016 [46]	Weekly Steps	Smartphone and Smartwatch Accelerometers	3 weeks	No significant PA results	Between-Group
Fiedler et al., 2023 [47]	Step Count, METs	Move 3/Move 4, or Movisens GmbH Accelerometer	3 weeks	Increased step count in “engaged” condition vs. “not engaged” (Δ: +256 steps)	Within-Person
Golbus, Shi et al., 2024 [34]	Step Count	iPhone or Android Phone and Apple Watch Series 4 or Fitbit Versa 2	6 months	Increased step count in Fitbit group during initiation phase only (Δ: +17%)	Within-Person
Hiremath et al., 2019 [48]	Energy Expenditure (kcal), Light PA Time, MVPA Time	Nexus 5 or 5X Smartphone, LG-Urbane Smartwatch, PanoBike Wheel Rotation Monitor	3 months	No significant PA results	Within-Person
Ismail et al., 2022 [49]	Step Count, METs	Android Smartphone	66 days	No significant PA results	Between-Group
Klasnja et al., 2019 [50]	Steps	Android Smartphone, Jawbone Smartwatch	6 weeks	No significant PA results	Within-Person
Low et al., 2023 [51]	Step Count	Fitbit Versa Smartwatch, Google Pixel 2 Smartphone	Varied based on surgery and discharge dates	No significant PA results	Within-Person
Pellegrini et al., 2021 [42]	% of Time Performing Light PA or MVPA	Shimmer, Intervention Accelerometer	1 month	Increased light PA (Δ: +7.8%)	Within-Person
Rabbi et al., 2015 [52]	Minutes per Day Walking	Self-Report	3 weeks	Increased MVPA (Δ: +10.4 min/day)	Between-Group
Sporrel et al., 2022 [40]	Minutes of Behavior	Actigraph Accelerometer	4 weeks	No significant PA results	Between-Group, then Within-Person
Thomas & Bond, 2015 [53]	Walking Time	SenseWear Mini Armband	3 weeks	3 min condition (Δ: +31 min/day) had a greater increase in light PA compared to 12 min (Δ: +15.3 min/day)	Between-Group
van Dantzig et al., 2013—Study 2 [43]	Proportion of Active Minutes	Accelerometer	6 weeks	No significant PA results	Between-Group
Wunsch et al., 2024 [54]	Steps/Week, MVPA Time/Week	Move 3, Move 4, or Movisens Accelerometer	3 weeks	No significant PA results	Between-Group

kcal = kilocalories; MVPA = moderate-to-vigorous physical activity; METs = metabolic equivalents; PA = physical activity.

## Abbreviations

The following abbreviations are used in this manuscript:

AI	Artificial intelligence
BCT	Behavior change technique
CI	Confidence interval
COM-B	Capability, opportunity, motivation–behavior
JITAI	Just-in-time adaptive intervention
mERA	Mobile health evidence reporting and assessment
METs	Metabolic equivalents
mHealth	Mobile health
MVPA	Moderate-to-vigorous physical activity
PA	Physical activity

## Appendix A

**Table A1:** PRISMA extension for scoping reviews (PRISMA-ScR): checklist and explanation.

Section	Item	PRISMA-ScR Checklist Item	Reported on Page #
TITLE			
Title	1	Identify the report as a scoping review.	1
ABSTRACT			
Structured summary	2	Provide a structured summary that includes (as applicable): background, objectives, eligibility criteria, sources of evidence, charting methods, results, and conclusions that relate to the review questions and objectives.	1
INTRODUCTION			
Rationale	3	Describe the rationale for the review in the context of what is already known. Explain why the review questions/objectives lend themselves to a scoping review approach.	1–2
Objectives	4	Provide an explicit statement of the questions and objectives being addressed with reference to their key elements (e.g., population or participants, concepts, and context) or other relevant key elements used to conceptualize the review questions and/or objectives.	2
METHODS			
Protocol and registration	5	Indicate whether a review protocol exists; state if and where it can be accessed (e.g., a Web address); and if available, provide registration information, including the registration number.	N/A
Eligibility criteria	6	Specify characteristics of the sources of evidence used as eligibility criteria (e.g., years considered, language, and publication status), and provide a rationale.	3
Information sources *	7	Describe all information sources in the search (e.g., databases with dates of coverage and contact with authors to identify additional sources), as well as the date the most recent search was executed.	2–4
Search	8	Present the full electronic search strategy for at least 1 database, including any limits used, such that it could be repeated.	Appendix B
Selection of sources of evidence †	9	State the process for selecting sources of evidence (i.e., screening and eligibility) included in the scoping review.	2–4
Data charting process ‡	10	Describe the methods of charting data from the included sources of evidence (e.g., calibrated forms or forms that have been tested by the team before their use, and whether data charting was performed independently or in duplicate) and any processes for obtaining and confirming data from investigators.	N/A
Data items	11	List and define all variables for which data were sought and any assumptions and simplifications made.	3
Critical appraisal of individual sources of evidence §	12	If necessary, provide a rationale for conducting a critical appraisal of included sources of evidence; describe the methods used and how this information was used in any data synthesis (if appropriate).	N/A
Synthesis of results	13	Describe the methods of handling and summarizing the data that were charted.	3–4
RESULTS			
Selection of sources of evidence	14	Give numbers of sources of evidence screened, assessed for eligibility, and included in the review, with reasons for exclusions at each stage, ideally using a flow diagram.	4
Characteristics of sources of evidence	15	For each source of evidence, present characteristics for which data were charted and provide the citations.	4–5
Critical appraisal within sources of evidence	16	If necessary, present data on critical appraisal of included sources of evidence (see item 12).	N/A
Results of individual sources of evidence	17	For each included source of evidence, present the relevant data that were charted that relate to the review questions and objectives.	5–10
Synthesis of results	18	Summarize and/or present the charting results as they relate to the review questions and objectives.	5–10
DISCUSSION			
Summary of evidence	19	Summarize the main results (including an overview of concepts, themes, and types of evidence available), link to the review questions and objectives, and consider the relevance to key groups.	10–12
Limitations	20	Discuss the limitations of the scoping review process.	12
Conclusions	21	Provide a general interpretation of the results with respect to the review questions and objectives, as well as potential implications and/or next steps.	12
FUNDING			
Funding	22	Describe sources of funding for the included sources of evidence, as well as sources of funding for the scoping review. Describe the role of the funders of the scoping review.	12

JBI = Joanna Briggs Institute; PRISMA-ScR = Preferred Reporting Items for Systematic reviews and Meta-Analyses extension for Scoping Reviews. * Where sources of evidence (see second footnote) are compiled from, such as bibliographic databases, social media platforms, and Web sites. † A more inclusive/heterogeneous term used to account for the different types of evidence or data sources (e.g., quantitative and/or qualitative research, expert opinion, and policy documents) that may be eligible in a scoping review as opposed to only studies. This is not to be confused with information sources (see first footnote). ‡ The frameworks by Arksey and O’Malley (6) and Levac and colleagues (7) and the JBI guidance (4, 5) refer to the process of data extraction in a scoping review as data charting. § The process of systematically examining research evidence to assess its validity, results, and relevance before using it to inform a decision. This term is used for items 12 and 19 instead of “risk of bias” (which is more applicable to systematic reviews of interventions) to include and acknowledge the various sources of evidence that may be used in a scoping review (e.g., quantitative and/or qualitative research, expert opinion, and policy document).

## Appendix B

**Table A2:** Search strategy for PsycINFO (EBSCO).

Search	Terms	Results
S1	DE “Digital Health Resources” OR DE “Digital Interventions” OR DE “Mobile Phones” OR DE “Smartphones” OR DE “Mobile Health Applications” OR DE “Mobile Applications” OR DE “Mobile Health” OR DE “Mobile Devices” OR DE “Mobile Technology” OR DE “Wearable Devices”	19,129
S2	TI (mhealth OR ehealth OR “digital health” OR “electronic health”) OR AB (mhealth OR ehealth OR “digital health” OR “electronic health”) OR KW (mhealth OR ehealth OR “digital health” OR “electronic health”)	9367
S3	TI (((cyber OR digital OR remote* OR distance* OR phone* OR internet OR “smart phone*” OR smartphone* OR mobile* OR iPhone* OR Android*) N5 (based OR app OR apps OR application* OR health OR intervention* OR delivery))) OR AB (((cyber OR digital OR remote* OR distance* OR phone* OR internet OR “smart phone*” OR smartphone* OR mobile* OR iPhone* OR Android*) N5 (based OR app OR apps OR application* OR health OR intervention* OR delivery))) OR KW (((cyber OR digital OR remote* OR distance* OR phone* OR internet OR “smart phone*” OR smartphone* OR mobile* OR iPhone* OR Android*) N5 (based OR app OR apps OR application* OR health OR intervention* OR delivery)))	37,861
S4	TI (((“activit* track*” OR “fitness track*” OR Fitbit OR Garmin OR TomTom OR Jawbone OR Withings OR “Apple Watch” OR smartwatch OR “smart watch” OR Amazfit OR “Google Pixel” OR “Galaxy Watch”))) OR AB (((“activit* track*” OR “fitness track*” OR Fitbit OR Garmin OR TomTom OR Jawbone OR Withings OR “Apple Watch” OR smartwatch OR “smart watch” OR Amazfit OR “Google Pixel” OR “Galaxy Watch”))) OR KW (((“activit* track*” OR “fitness track*” OR Fitbit OR Garmin OR TomTom OR Jawbone OR Withings OR “Apple Watch” OR smartwatch OR “smart watch” OR Amazfit OR “Google Pixel” OR “Galaxy Watch”)))	1197
S5	S1 OR S2 OR S3 OR S4	53,154
S6	TI (((“Just-in-time” OR “just in time” OR “just-in-time adaptive intervention*” OR JITAI OR “adaptive intervention*”) OR (“ecologic* momentary intervention*” OR EMI) OR (“experience sampling” OR “ambulatory assess*” OR “moment-to-moment measures” OR “daily diary” OR “repeat* assessment*” OR “ambulatory monitor*” OR “electronic diar*”) OR (“real time intervention*” OR “context* aware*” OR “context* trigger*” OR “context* tailor*” OR “dynamic tailor*” OR “real time tailor*” OR “real time intervention” OR “real time therapy” OR “real time tailor*” OR “sensor triggered” OR geofenc* OR “context* sens*” OR “real time context*” OR “persuasive technolog*” OR “sensing technolog*”))) OR AB (((“Just-in-time” OR “just in time” OR “just-in-time adaptive intervention*” OR JITAI OR “adaptive intervention*”) OR (“ecologic* momentary intervention*” OR EMI) OR (“experience sampling” OR “ambulatory assess*” OR “moment-to-moment measures” OR “daily diary” OR “repeat* assessment*” OR “ambulatory monitor*” OR “electronic diar*”) OR (“real time intervention*” OR “context* aware*” OR “context* trigger*” OR “context* tailor*” OR “dynamic tailor*” OR “real time tailor*” OR “real time intervention” OR “real time therapy” OR “real time tailor*” OR “sensor triggered” OR geofenc* OR “context* sens*” OR “real time context*” OR “persuasive technolog*” OR “sensing technolog*”))) OR KW (((“Just-in-time” OR “just in time” OR “just-in-time adaptive intervention*” OR JITAI OR “adaptive intervention*”) OR (“ecologic* momentary intervention*” OR EMI) OR (“experience sampling” OR “ambulatory assess*” OR “moment-to-moment measures” OR “daily diary” OR “repeat* assessment*” OR “ambulatory monitor*” OR “electronic diar*”) OR (“real time intervention*” OR “context* aware*” OR “context* trigger*” OR “context* tailor*” OR “real time tailor*” OR “real time intervention” OR “real time therapy” OR “real time tailor*” OR “dynamic tailor*” OR “sensor triggered” OR geofenc* OR “context* sens*” OR “real time context*” OR “persuasive technolog*” OR “sensing technolog*”)))	13,332
S7	TI ((“daily life” or “real-time”) N5 (intervention* OR aware* OR triggered OR tailor* OR sens* OR *measure* OR assessment* OR test* OR monitor*)) OR AB (((“daily life” or “real-time”) N5 (intervention* OR aware* OR triggered OR tailor* OR sens* OR *measure* OR assessment* OR test* OR monitor*))) OR KW ((“daily life” or “real-time”) N5 (intervention* OR aware* OR triggered OR tailor* OR sens* OR *measure* OR assessment* OR test* OR monitor*))	4540
S8	S6 OR S7	17,375
S9	DE “Physical Fitness” OR DE “Aerobic Exercise” OR DE “Running” OR DE “Walking” OR DE “Physical Activity” OR DE “Weightlifting” OR DE “Exercise” OR DE “Locomotion” OR DE “Active Living”	78,438
S10	TI (“ physical activit*” OR “physical training” OR walk* OR exercis* OR fitness OR “physical fitness*” OR aerobics OR “aerobic* training” OR “aerobic* activit*” OR running OR jogging OR athletics OR cycling OR bike* OR biking OR bicycl* OR swim* OR hiking OR rollerblading OR roller-blading OR rollerskat* OR roller-skat* OR skating OR “physical exertion*” OR “active transport*” OR “circuit training” OR “strength training” OR calisthenics OR sport* OR “resistance training” OR “weight training” OR “endurance training” OR sport* OR pilates OR yoga) OR AB (“ physical activit*” OR “physical training” OR walk* OR exercis* OR fitness OR “physical fitness*” OR aerobics OR “aerobic* training” OR “aerobic* activit*” OR running OR jogging OR athletics OR cycling OR bike* OR biking OR bicycl* OR swim* OR hiking OR rollerblading OR roller-blading OR rollerskat* OR roller-skat* OR skating OR “physical exertion*” OR “active transport*” OR “circuit training” OR “strength training” OR calisthenics OR sport* OR “resistance training” OR “weight training” OR “endurance training” OR sport* OR pilates OR yoga) OR KW (“ physical activit*” OR “physical training” OR walk* OR exercis* OR fitness OR “physical fitness*” OR aerobics OR “aerobic* training” OR “aerobic* activit*” OR running OR jogging OR athletics OR cycling OR bike* OR biking OR bicycl* OR swim* OR hiking OR rollerblading OR roller-blading OR rollerskat* OR roller-skat* OR skating OR “physical exertion*” OR “active transport*” OR “circuit training” OR “strength training” OR calisthenics OR sport* OR “resistance training” OR “weight training” OR “endurance training” OR sport* OR pilates OR yoga)	239,890
S11	S9 OR S10	249,104
S12	S5 AND S8 AND S11	222

## Appendix C

**Table A3:** Study extraction table.

Author and Year	Location	Design	Sample Size	Participant Characteristics	Behavior Change Theory	BCTs	JITAI Description
Bardus et al., 2018 [33]	Lebanon	Protocol	Recruitment has not commenced	Healthy adult employees of an Academic Institution	Not Stated	Self-monitoring of behavior *; goal-setting (behavior) *; feedback on behavior *; social reward *; social support *	Mobile coach that provides interactive counseling and feedback based on several outcomes
Boerema et al., 2019 [45]	The Netherlands	Quasi-experimental	*n* = 14	50-year-old+ office workers who spend 50% of their time or more at a computer	Not Stated	Prompts/cues; goal-setting (behavior); feedback on behavior	Focuses on reducing sedentary behavior with periodic prompts
Carey et al., 2024 [31]	The United States	Protocol	*n* = 196	18-to-75-year-olds with a traumatic SCI injury at C5 and below who are 6 months+ post injury and use a wheelchair as their primary means of transportation	COM-B Model	Goal-setting (behavior) *; feedback on behavior *; prompts/cues *;	Micro-randomized feedback prompts and activity recommendations
Coppens et al., 2024 [16]	Belgium	Randomized Controlled Trial	*n* = 25	Healthy, inactive adults	Self-Determination Theory	Prompts/cues *; instruction on how to perform the behavior	Uses machine learning for personalized activity suggestions
Daryabeygi-Khotbehsara et al., 2023 [35]	Australia	Protocol	Recruitment has not commenced	Adults between 35 and 65 years old with type 2 diabetes	Not Stated	Behavior substitution *; social support *; problem-solving *; instruction on how to perform the behavior *; information about health consequences *; prompts/cues *; goal-setting (behavior); self-monitoring of behavior; feedback on behavior *	Notifications to encourage either sedentary interruption or movement
Ding et al., 2016 [46]	The United States	Feasibility Trial	Total *n* = 16, control *n* = 7, intervention *n* = 9	College students	Fogg Behavior Model; Locke and Latham’s Goal-Setting Theory; Trans-theoretical Model	Goal-setting (behavior) *; prompts/cues *	Reminders were sent when participants overused their smartphone, were sedentary for extended periods, were walking (to encourage more walking), or had just finished a meal
Fiedler et al., 2023 [47]	Germany	Randomized Controlled Trial	*n* = 80	Families including at least one parent and at least one child who was 10 years of age or older and who were living together in a common household	Self-Determination Theory	Goal-setting (behavior) *; prompts/cues *; instruction on how to perform the behavior	Notifications to disrupt bouts of sedentary behavior (50/60 min of the last hour)
Golbus et al., 2024a [32]	United States	Development Study	*n* = 108	Adults 18 to 75 years old enrolled in cardiac rehabilitation	Not Stated	Goal-setting (behavior), Self-monitoring of behavior, Prompts/cues	Physical activity notifications are designed to disrupt sedentary behavior (e.g., to stand up or stretch) and to encourage low-level physical activity (e.g., bouts of 250 to 500 steps).
Golbus et al., 2024b [34]	United States	Micro-randomized Trial	Recruitment has not commenced	Adults with self-reported hypertension and no contradictions to PA or a low-sodium diet	Not Stated	Goal-setting (behavior) *, Prompts/cues *, Visualizations *, Feedback on behavior *	Static component (e.g., a mobile application that allowed for goal-setting and activity-tracking) and a dynamic component that consisted of micro-randomized text messages
Hietbrink et al., 2023 [36]	The Netherlands	Development Study	*n* = 9	Adults with type 2 diabetes	HAPA Model; Rothman’s Theory; Marlatt’s Relapse-Prevention Theory	Goal-setting (behavior) *; problem-solving *; action planning *; review behavior goal *; feedback on behavior *; self-monitoring of behavior *; self-monitoring of outcomes of behavior *; social support *; social support (practical) *; instruction on how to perform the behavior *; information about health consequences *; information about emotional consequences *; information about others’ approval *; prompts/cues *; habit formation *; credible source *; pros and cons *; comparative imagining of future outcomes *; reduce negative emotions *; framing and reframing *; verbal persuasion about capability *; focus on past success *	425 motivational messages, consisting of content for each of the tailoring variables; decision points took place at two semirandom times per day
Hiremath et al., 2019 [48]	The United States	Pilot Study	*n* = 16	Adults 18 to 65 years old with an SCI who are 6 months+ post injury and use a wheelchair as their primary means of transportation	Social Cognitive Theory	Self-monitoring of behavior; feedback on behavior; goal-setting (behavior); instruction on how to perform the behavior	Provided proactive, near-real-time feedback via smartphone (audio/vibration, based on user preference) and smartwatch (vibration) when participants engaged in moderate- or higher-intensity physical activity
Hojjatnia et al., 2021 [67]	The United States	Quasi-experimental	*n* = 45	Insufficiently active emerging and young adults (18–29 years old)	Not Stated	Prompts/cues	Messages were sourced from three libraries (move more, sit less, inspirational quotes); smartphone location data were used to retrieve local weather at message delivery, and system identification and simulations evaluated responses to each message type under varying conditions, with extracted features summarizing dynamic response patterns
Ismail et al., 2022 [49]	Qatar	Quasi-experimental	Total *n* = 58, control *n* = 29, intervention *n* = 29	Sedentary adult employees aged 23 to 39 years old	Not Stated	Prompts/cues; material reward (behavior); self-monitoring (behavior)	Personalized prompts that include user information, user goals, daily routine, and the surrounding environment
Klasnja et al., 2019 [50]	The United States	Micro-randomized Trial	*n* = 44	18- to 60-year-old adults with a regular schedule outside the home (employed or a student)	Not Stated	Prompts/cues; self-monitoring of behavior	Contextually tailored activity suggestions to walk (intended to encourage bouts of 500–1000 steps) and suggestions to disrupt sedentary behavior (to stand up, stretch, and/or move around)
Kramer et al., 2019 [39]	Switzerland	Protocol	*n* = 274	Healthy adults not working night shifts	Not Stated	Problem-solving *; action planning *; discrepancy between current behavior and goal *; feedback on behavior *; instruction on how to perform the behavior *; material reward (behavior) *; nonspecific reward *; self-monitoring of behavior *; prompts/cues *	Sent out intervention and step goal-related notifications at random points in time but within prespecified time windows that guaranteed delivery at appropriate times
Low et al., 2023 [51]	The United States	Pilot Study	Total *n* = 26, control *n* = 13, intervention *n* = 13	Patients scheduled for surgery for metastatic gastrointestinal cancer	Not Stated	Prompts/cues	Real-time mobile intervention that detects prolonged sedentary behavior during the perioperative period and delivers walking prompts tailored to daily self-reported symptoms
Mair et al., 2023 [41]	Singapore	Feasibility Study	*n* = 31	Older adults who use a smartphone	COM-B Model	Goal-setting (behavior) *; action planning *; prompts/cues *; self-monitoring of behavior *; feedback on behavior *	Personalized PA messaging was delivered by the JitaBug app to encourage participants to meet their daily PA goal; messages were tailored to participants’ contexts
Novak et al., 2024 [68]	The Czech Republic	Development	*n* = 14	Adults with prediabetes	Self-regulation Theory	Prompts/cues *; habit formation *; behavior substitution *; review behavior goal *; discrepancy between current behavior and goal *; feedback on behavior *; social reward *; action planning *; information about health consequences *	“Walk faster” notifications while walking and “stand up” notifications after prolonged sitting
Park et al., 2023 [56]	The United States	Protocol	*n* = 48	Inactive adults aged 25 years or older	Social Cognitive Theory	Prompts/cues *; goal-setting (behavior) *; feedback on behavior *	
Pellegrini et al., 2021 [42]	The United States	Feasibility Study	*n* = 8	Adults aged 21 to 70 years old with type 2 diabetes	Not Stated	Prompts/cues *	Using an external accelerometer (Shimmer), when a person is sedentary for ≥20 min, they received a prompt to engage in light behavior for 2 min
Rabbi et al., 2015 [52]	The United States	Randomized Controlled Trial	Total *n* = 17, control *n* = 8, intervention *n* = 9	Low to moderately active adults	Learning Theory; Social Cognitive Theory; Fogg’s Behavioral Model	Self-monitoring of behavior; prompts/cues	Used auto/manual logging of activity, location, and food; analyzed logs to detect behavior patterns; applied multi-armed bandit algorithm to deliver personalized suggestions (continue, avoid, or modify behaviors)
Sporrel et al., 2022 [40]	The Netherlands	Feasibility Study	Total *n* = 20, basic intervention *n* = 9, smart intervention *n* = 11	Adults aged 18 to 55 years old who would like to become more active	Fogg’s Behavioral Model	Goal-setting (behavior) *; review behavioral goals *; self-monitoring of behavior *; instruction on how to perform the behavior *; demonstration of the behavior *; behavioral practice/rehearsal *; graded tasks *; situation-specific reward *; prompts/cues	Compared two app versions: Basic PAUL (random-timed JIT prompts) vs. Smart PAUL (JIT adaptive prompts for walking/running)
Thomas & Bond, 2015 [53]	The United States	Quasi-experimental	*n* = 30	Overweight or obese men and women aged 21 to 70 years old	None Reported	Prompts/cues	Three conditions: (1) 3 min break after 30 min sedentary; (2) 6 min break after 60 min; (3) 12 min break after 120 min
van Dantzig et al., 2013 [43]	The Netherlands	Pilot Study	Total *n* = 86, control *n* = 46, intervention *n* = 40	Healthy adult office workers	Social Influence Strategies Defined by Cialdini	Prompts/cues *; goal-setting (behavior); feedback on the behavior; social support; habit formation	Not stated
Vandelanotte et al., 2023 [17]	Australia	Development Study	Recruitment has not commenced	Adults interested in becoming more physically active	Self-Determination Theory	Social support (unspecified); information about health consequences; action planning; habit formation; restructuring the physical environment; incentive (outcome); framing/reframing; prompts/cues	(1) NLP-based conversations to increase activity knowledge; (2) reinforcement learning-driven nudge engine using real-time data (activity, GPS, GIS, weather, user input); (3) generative AI Q&A for physical activity support
Vetrovsky et al., 2023 [37]	The Czech Republic	Protocol	Recruitment has not commenced	Adults aged 18 or older with a diagnosis of prediabetes or type 2 diabetes according to Czech guidelines	Not Stated	Credible source *; instruction on how to perform the behavior *; action planning *; information about health consequences *; graded tasks *; goal-setting (behavior) *; self-monitoring of behavior *; prompts/cues *; habit formation *; behavior substitution *; review behavioral goals *; discrepancy between current behavior and goal *; feedback on behavior *; social reward *; information about health consequences *; problem-solving *; social support (unspecified) *; graded tasks *	Just-in-time prompts to increase walking pace were triggered when the patient walked for 5 consecutive minutes with an average steps per minute ranging between 60 and 100; just-in-time prompts to interrupt sitting were sent when the patient sits for more than 30 min
Vos et al., 2023 [38]	The Netherlands	Pilot Study	*n* = 11	Adults aged 45 years or older	Social Cognitive Theory	Action planning *; social reward *; feedback on behavior *; social comparison *; information about health consequences *; instruction on how to perform the behavior *; goal-setting (behavior) *; self-monitoring of behavior *; review of outcome goals *; prompts/cues	Not stated
Wang et al., 2021 [69]	The Netherlands	Feasibility Study	*n* = 7	Adults struggling to maintain a healthy activity level who would like to be more physically active	Behavior Change Wheel	Prompts/cues *	The reinforcement learning model would track user data and take input from physical activity logs to determine if it was the right time to send a prompt
Wunsch et al., 2024 [54]	Germany	Randomized Controlled Trial	Total *n* = 156, control *n* = 68, intervention *n* = 88	Households comprising at least one adult caregiver and one child aged >10 years residing together	Not Stated	Goal-setting (behavior) *; feedback on behavior *; self-monitoring of behavior *; instruction on how to perform the behavior; social comparison *; prompts/cues	Not stated

BCTs = behavior change techniques; PA = physical activity. BCTs denoted with a * were explicitly stated by the author.
